# Hemodynamic analysis of the aortic root based on differences in outflow graft angle in patients with implantable left ventricular assist devices

**DOI:** 10.1007/s10047-026-01547-w

**Published:** 2026-03-03

**Authors:** Hiroaki Yamamoto, Hiroki Kohno, Hiroki Ikeuchi, Tomoyoshi Kanda, Michiko Watanabe, Tomohiko Inui, Kaoru Matsuura, Goro Matsumiya

**Affiliations:** Department of Cardiovascular Surgery, Chiba University Hospital, 1-8-1, Inohana, Chuo-ku, Chiba, 260-8677 Japan

**Keywords:** LVAD, Aortic insufficiency, Wall shear stress, CFD, Coronary blood flow, Outflow graft

## Abstract

Aortic insufficiency (AI) that develops during long-term support with continuous-flow left ventricular assist devices (LVADs) remains a significant clinical problem. Abnormal flow patterns and altered wall shear stress (WSS) on the aortic valve are believed to contribute to leaflet remodeling, yet the hemodynamic influence of outflow graft orientation in patient-specific anatomies is not fully understood. This study investigated how different outflow graft angles affect aortic valve WSS and coronary perfusion using computational fluid dynamics (CFD). Three LVAD recipients who underwent preoperative ECG-gated coronary CT were retrospectively analyzed. Patient-specific geometries of the ascending aorta, aortic root, and coronary arteries were reconstructed. A cylindrical outflow graft was virtually anastomosed 25 mm above the sino-tubular junction, and three inclination angles (30°, 60°, 90°) were modeled with a fixed azimuthal angle of 90°. CFD simulations were performed under constant LVAD inflow (5.0 L/min) and uniform outlet pressure conditions. WSS on the aortic valve cusps and coronary flow rates were evaluated after achieving quasi-steady flow. Increasing outflow graft angle resulted in higher WSS on the ascending aortic wall opposite the anastomosis and consistently elevated WSS on the left coronary cusp. In some cases, the non-coronary cusp also showed localized WSS increases. Coronary flow decreased with shallower graft angles, with left coronary artery flow approximately halved at 30° compared with 90°, while right coronary flow exhibited a smaller reduction. Outflow graft angle substantially affects aortic valve WSS distribution and coronary perfusion. Steeper angles increase leaflet WSS, whereas shallower angles reduce coronary flow. Patient-specific CFD simulations may aid in optimizing graft positioning and reducing the risk of AI progression in LVAD patients.

## Introduction

Implantable left ventricular assist devices (LVADs) are widely used as bridge-to-transplantation therapy for patients with advanced heart failure awaiting heart transplantation, as well as destination therapy for those who are not transplant candidates due to advanced age or comorbidities [1]. In Japan, the severe donor shortage has resulted in a progressive prolongation of the waiting period for heart transplantation, leading to an increasing number of patients supported by LVADs for extended durations [2]. Consequently, complications that arise during prolonged LVAD support—such as aortic valve insufficiency (AI) and right heart failure—have emerged as major determinants of long-term outcomes [3].

De novo or progressive AI that develops during LVAD support is known to cause reduced effective cardiac output, left ventricular dilation, hemolysis, and recurrent heart failure, and is associated with poor prognosis [3]. Although surgical interventions—including aortic valve closure, replacement, and repair—may be considered, consensus has not yet been established regarding optimal procedural strategy or indications [4].

The pathophysiology of LVAD-associated AI is thought to involve abnormal flow patterns and altered wall shear stress (WSS) acting on the aortic valve due to chronic valve non-opening. However, few studies have quantitatively evaluated the hemodynamic effects of outflow graft geometry and anastomotic angle on the aortic valve using computational fluid dynamics (CFD).

The aim of this study was to construct patient-specific aortic root models from LVAD recipients and numerically investigate the influence of different LVAD outflow graft angles on WSS exerted on the aortic valve. Through this approach, we sought to identify graft configurations that minimize adverse hemodynamic loading on the valve.

## Methods

**Fig. 1:**
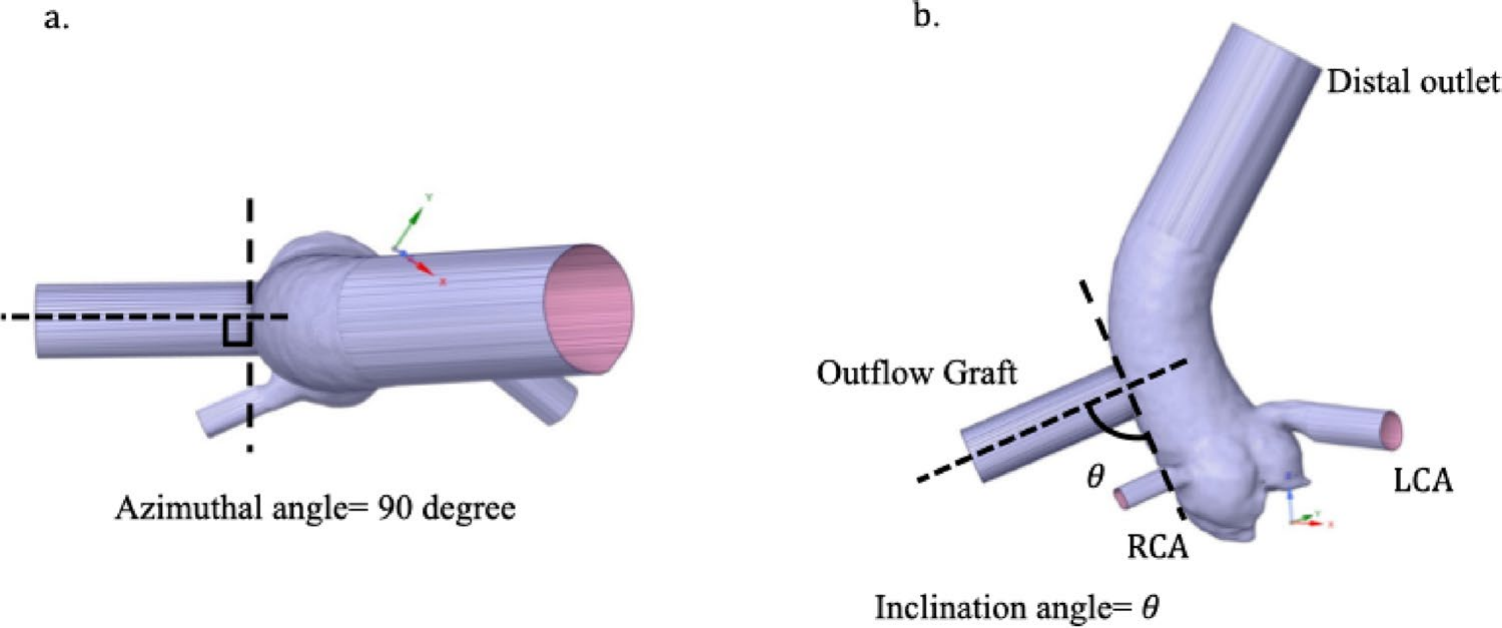
Outflow graft orientation. **a** Definition of the azimuthal angle, which was fixed at 90°. **b** Definition of the inclination angle (θ), varied at 30°, 60°, and 90°, illustrating the relationship between the outflow graft, the ascending aorta, and the coronary arteries (RCA and LCA). “Distal outlet” indicates the distal ascending aorta boundary.

### Study design

Among patients who underwent HeartMate 3 left ventricular assist device (Abbott Laboratories, Abbott Park, IL, USA) implantation at our institution as bridge-to-transplantation therapy, three patients who had undergone preoperative ECG-gated coronary computed tomography (CT) were retrospectively selected. Patients with ischemic cardiomyopathy were excluded. This retrospective study was approved by the institutional review board of our hospital (Protocol Nos. 1250 and 3507; approval dates: June 26, 2017, and January 23, 2020, respectively). The requirement for individual informed consent was waived.

### Patient-specific aortic root and coronary geometry

All patients underwent ECG-gated coronary CT using a 320-detector row system (Aquilion ONE; Toshiba Medical Systems, Otawara, Japan) at 120–135 kV. Images were reconstructed with 0.5-mm slice thickness. Three-dimensional patient-specific geometries of the ascending aorta, aortic root, and coronary arteries were exported from Ziostation2 Plus software (version 2.9.8.0; Ziosoft, Tokyo, Japan) and reconstructed using ANSYS SpaceClaim (ANSYS Inc., Canonsburg, PA, USA). The aortic valve was assumed to be completely closed to represent continuous-flow LVAD support. After segmentation of the aortic root, right coronary artery (RCA), and left coronary artery (LCA), geometric smoothing and repair were performed to obtain a watertight computational domain.

The LVAD outflow graft was modeled as a cylindrical graft (14-mm diameter, 40-mm length) virtually anastomosed to the ascending aorta at a position 25 mm cranial to the sino-tubular junction (STJ), based on published anatomical reference distances and empirical surgical considerations. Three anastomotic angles—inclination angles of 30°, 60°, and 90°, each with a fixed azimuthal angle of 90°—were constructed (Fig. 1). In this study, the term “outflow graft angle” refers to the inclination angle. Apart from graft angle, all geometric parameters were identical among models. Each outlet region (distal aorta, RCA, LCA) was extended linearly to at least twice the vessel diameter to reduce numerical disturbances near the boundaries.

### Mesh generation

Meshes were generated using ANSYS Fluent Meshing (2022 R2). To accurately resolve WSS, unstructured hexahedral meshes with prism layers were created near vessel walls. A minimum of three prism layers with a growth ratio < 1.2 was applied. Mesh independence was confirmed by comparing flow rates and WSS distributions across successively refined meshes.

### Governing equations and blood properties

Blood flow was modeled as an incompressible Newtonian fluid governed by the three-dimensional unsteady Navier–Stokes equations. Blood density and viscosity were set to 1060 kg/m³ and 0.0035 Pa·s, respectively. Given the large vessel diameters and continuous-flow conditions, the Newtonian assumption was considered appropriate. Turbulence was assumed in all simulations.

## Boundary conditions

### Inlet (LVAD outflow)

A constant mass-flow inlet corresponding to 5.0 L/min (0.0883 kg/s) was prescribed at the outflow graft entrance, representing typical continuous-flow LVAD operation.

### Outlets

To avoid numerical instability associated with multi-element Windkessel models and to isolate the relative hemodynamic influence of outflow graft angle, constant static pressure conditions were applied:

Distal ascending aorta: 80 mmHg (10,665 Pa).

RCA outlet: 79 mmHg (10,532 Pa).

LCA outlet: 79 mmHg (10,532 Pa).

Coronary outlet pressures were implemented using a custom C-based user-defined function (UDF) compiled in ANSYS Fluent 2022 R2. While this simplified outlet strategy produces higher absolute coronary flows than physiologic resting values, it allows consistent comparison among graft angles.

### Wall boundary conditions

All vessel walls—including the aortic valve leaflets—were treated as rigid with no-slip conditions. The aortic valve remained fully closed throughout the simulation.

## Numerical simulation

Unsteady simulations were performed using ANSYS Fluent 2022 R2. The coupled scheme was used for pressure–velocity coupling, and second-order spatial discretization was applied. The k–ω turbulence model was employed. Based on numerical stability testing, the time step was set to 0.01 s, and simulations were continued for at least 6.0 s until quasi-steady flow was achieved. All computations were performed on a 16-core CPU in parallel.

### Wall shear stress and hemodynamic metrics

WSS on the aortic annulus and valve leaflets was calculated. As the flow reached a steady state, the WSS values at the end of the 6-second simulation were regarded as representative mean WSS values. The reported WSS corresponds to the peak value on each aortic valve leaflet.

### Coronary flow analysis

Mass-flow rates at the RCA, LCA, and distal aorta outlets were monitored. Time-averaged coronary flow and flow distribution ratios (RCA: LCA: distal aorta) were computed to evaluate the relative influence of graft angle on coronary perfusion.

### Comparative analysis

Simulations for the three outflow graft angles (30°, 60°, 90°) were conducted using identical inlet, outlet, and numerical conditions. WSS magnitude and spatial patterns on the aortic valve complex as well as coronary flow distribution were compared.

## Results

All three LVAD patients used in this study had dilated cardiomyopathy as the underlying diagnosis, and none showed preoperative coronary artery disease.

**Fig. 2:**
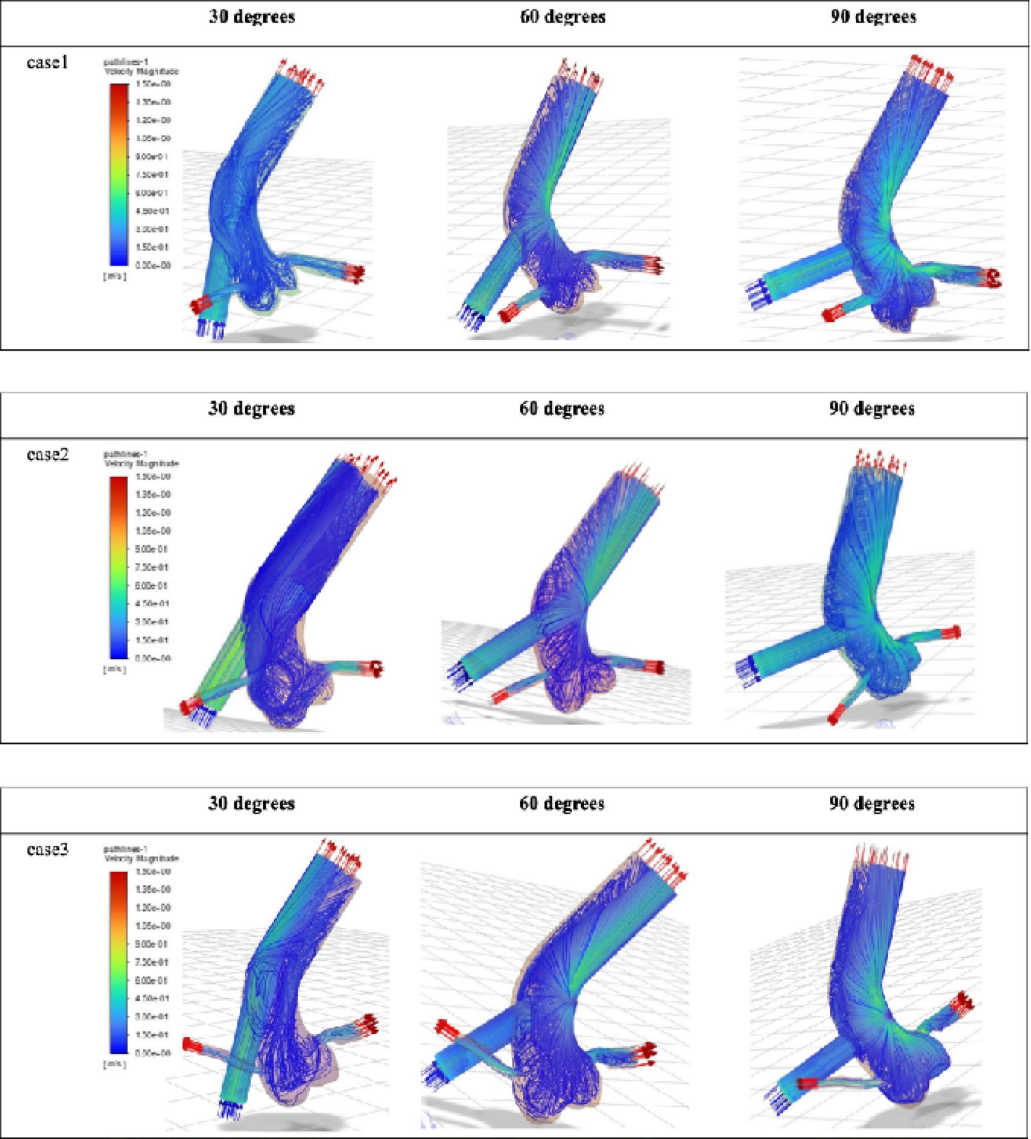
Velocity field and streamlines. Streamlines and color contour maps of velocity magnitude for the three patient-specific models at each outflow graft angle (30°, 60°, and 90°). Local acceleration of flow is observed particularly at higher graft angles.

**Fig. 3:**
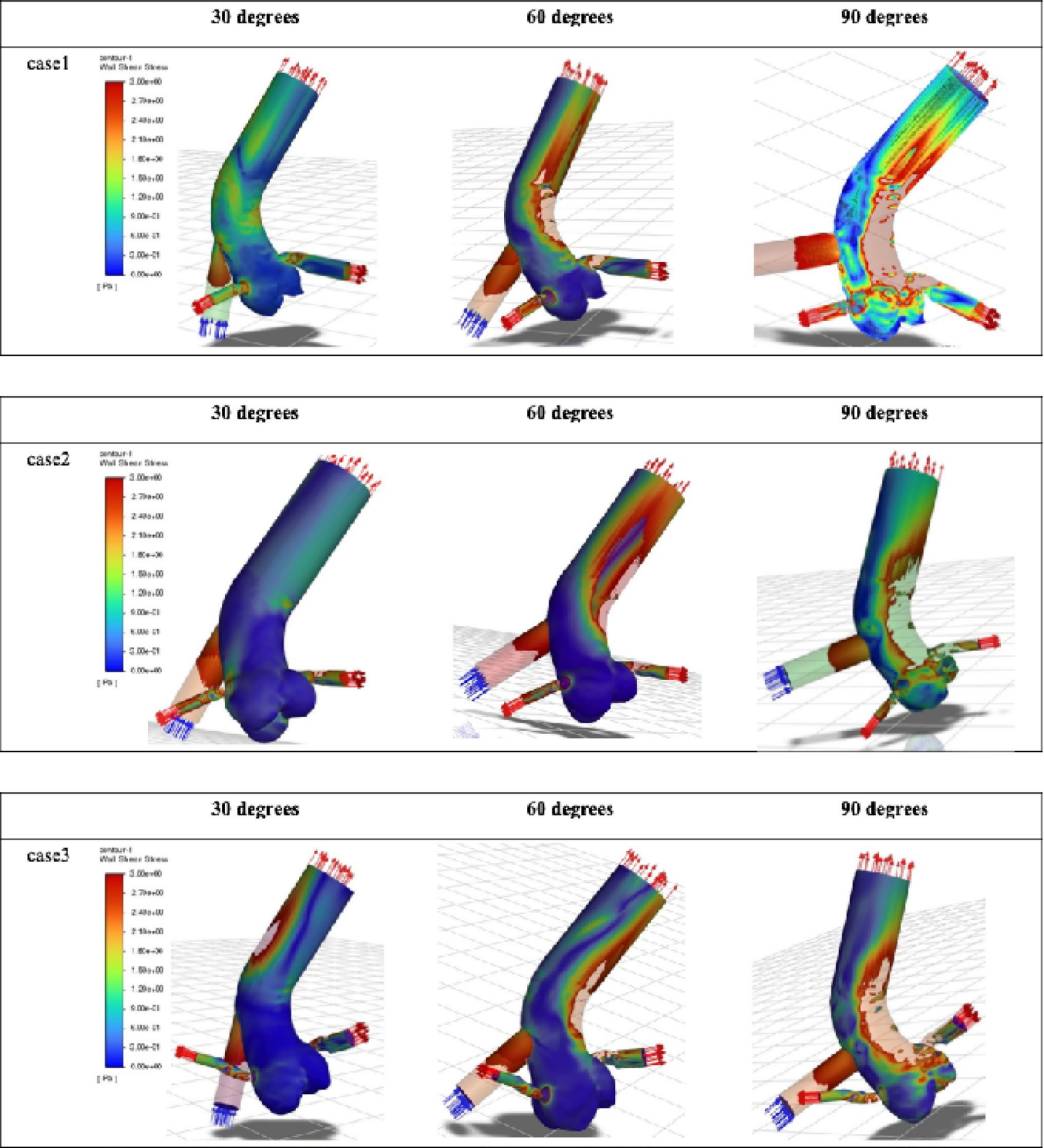
Wall shear stress distribution on the aortic wall. Color contour plots showing wall shear stress (WSS) on the ascending aorta and aortic root for all patients across the three outflow graft angles. Increased WSS is observed on the aortic wall opposite the outflow graft with increasing outflow graft angle.

**Fig. 4:**
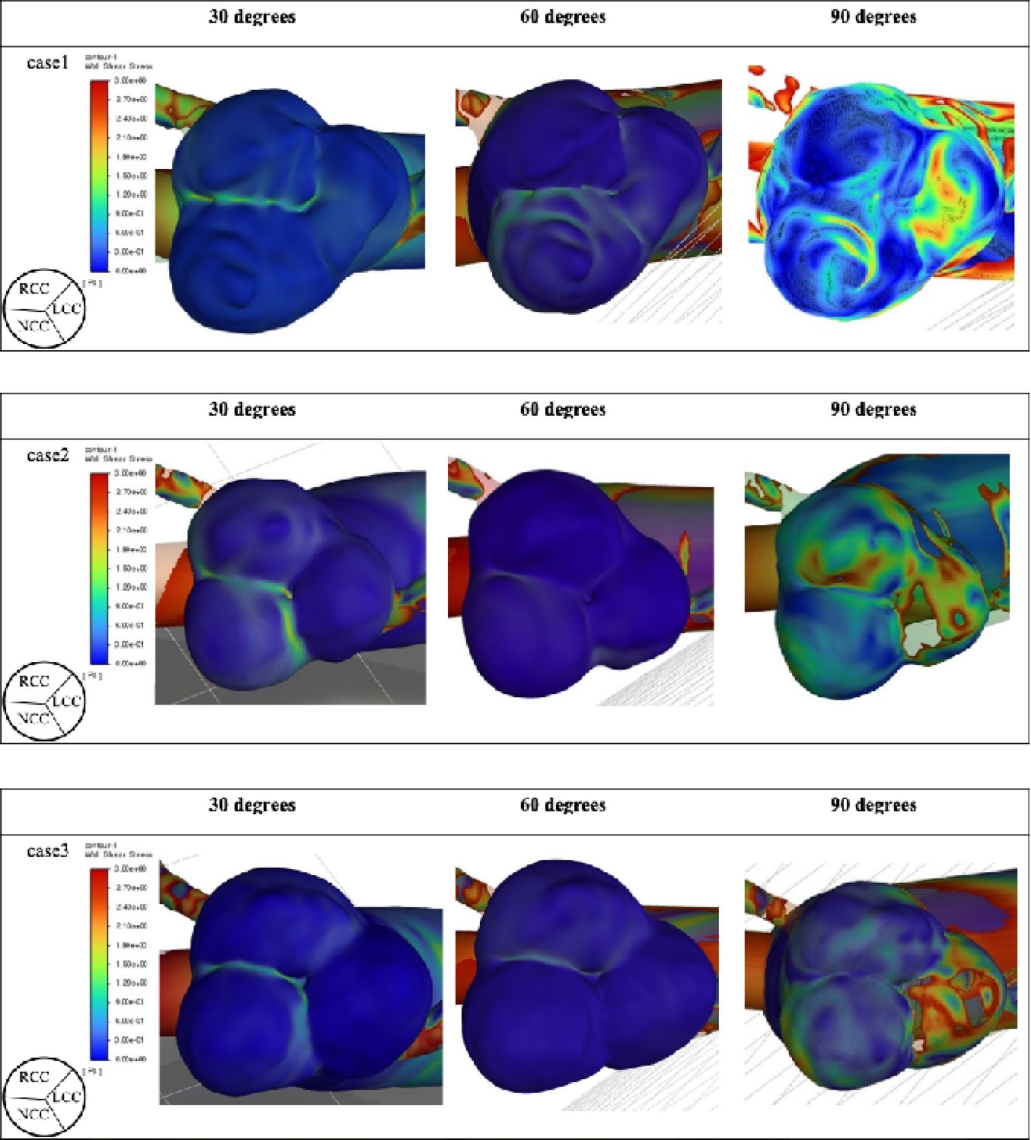
Wall shear stress on the aortic valve. WSS distribution on the aortic valve leaflets (RCC: right coronary cusp; LCC: left coronary cusp; NCC: non-coronary cusp) for each outflow graft angle in all patient models. Larger outflow graft angles (especially 90°) are associated with higher WSS, predominantly on the LCC.

**Fig. 5:**
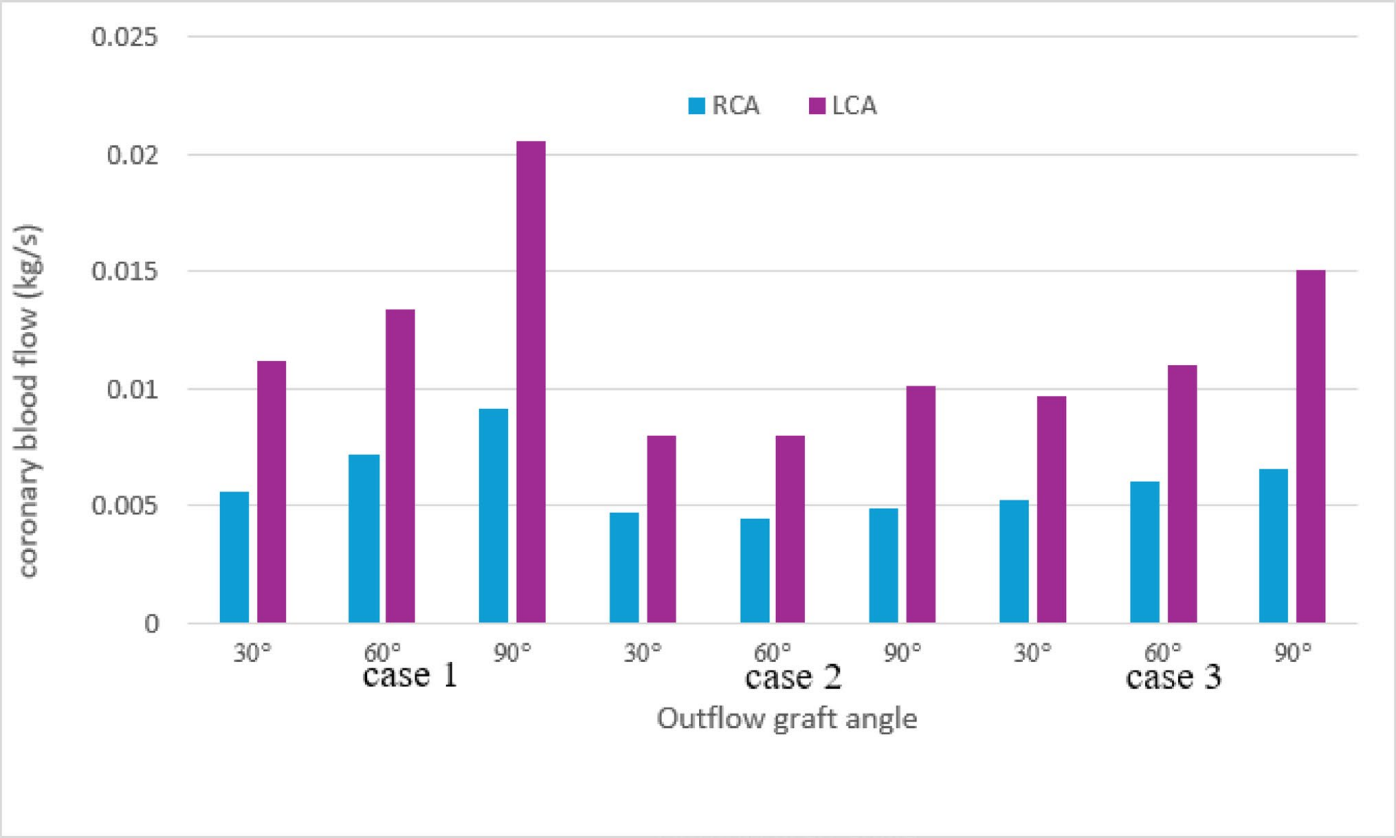
Coronary blood flow at each outflow graft angle. Time-averaged flow rates of the right coronary artery (RCA) and left coronary artery (LCA) for all three patient-specific models. LCA flow decreased progressively with shallower outflow graft angles, while RCA flow showed a similar but less pronounced tendency. RCA: right coronary artery; LCA: left coronary artery.

**Table 1:** Wall shear stress of the aortic cusps and mean blood velocity of coronary arteries

	WSS LCC(Pa)	WSS RCC(Pa)	WSS NCC(Pa)	Velocity RCA(m/s)	Velocity LCA(m/s)
Case1 30°	0.36	0.3	0.09	0.207	0.156
60°	0.33	0.059	0.059	0.265	0.189
90°	2.369	0.99	2.76	0.339	0.289
Case2 30°	0.059	0.27	0.63	0.249	0.257
60°	0.059	0.029	0.18	0.233	0.258
90°	5.8	2.66	0.63	0.259	0.330
Case3 30°	0.059	0.389	1.44	0.211	0.171
60°	0.9	0.33	0.02	0.243	0.195
90°	6.44	0.69	1.4	0.263	0.268

RCC Right coronary cusp LCC Left coronary cusp NCC Non-coronary cusp.

### Flow velocity

At an outflow graft angle of 90°, the outflow jet impinged on the opposite wall of the ascending aorta, resulting in a localized increase in flow velocity. A similar increase in velocity was also observed in the coronary arteries, particularly in the left coronary artery. In contrast, flow velocity within the aortic root showed minimal variation across the different outflow graft angles (Fig. 2).

**Fig. 6:**
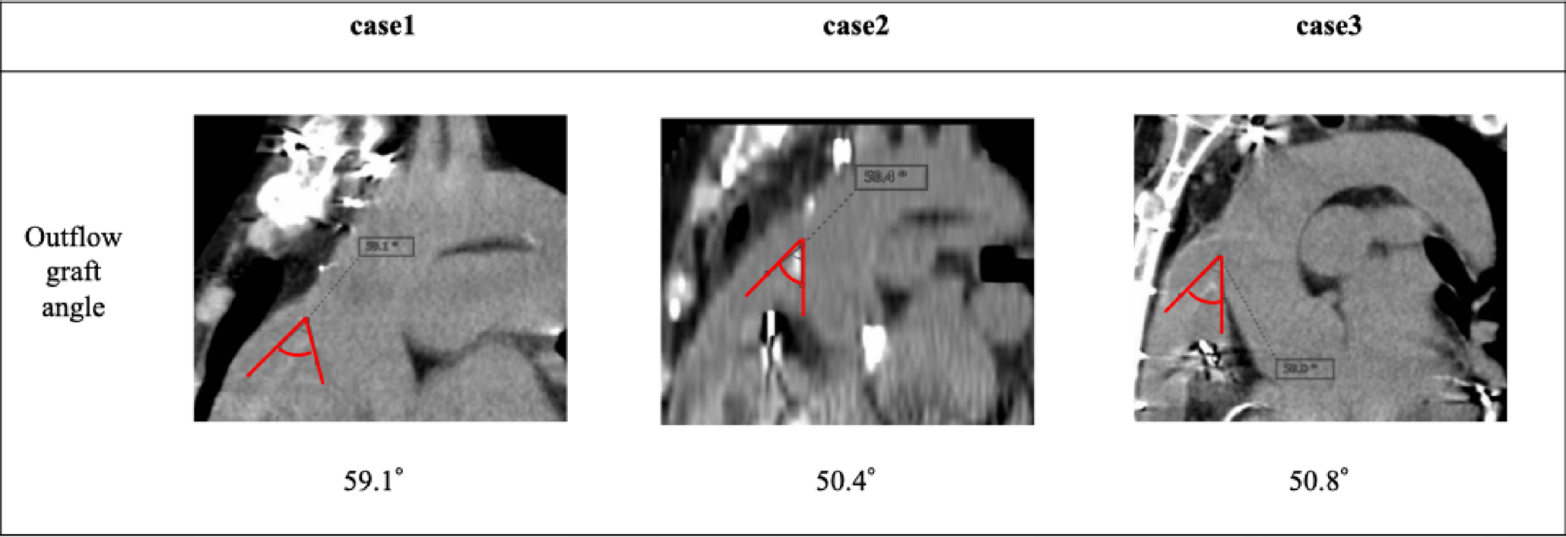
The actual outflow graft angles in Cases 1, 2, and 3 were 59.1°, 50.4°, and 50.8°, respectively

### Wall shear stress

Consistent with the velocity patterns, wall shear stress (WSS) increased on the ascending aortic wall opposite the graft anastomosis as the outflow graft angle increased (Fig. 3).

At the aortic valve level, the 90° configuration resulted in relatively high WSS on the left coronary cusp. In some cases, elevated WSS was also observed on the non-coronary cusp (Fig. 4; Table 1). No appreciable increase in WSS was identified in the region corresponding to the annulus.

### Coronary blood flow

A tendency toward reduced coronary blood flow was observed as the outflow graft angle became shallower. This reduction was particularly pronounced in the LCA; when comparing an outflow graft angle of 30° with that of 90°, a maximum decrease of approximately 50% was observed. In contrast, no substantial changes in RCA flow were noted among the different outflow graft angles (Fig. 5).

## Discussion

Using patient-specific CT-derived anatomies, we demonstrated that increasing outflow graft inclination angle consistently produced elevated WSS on the LCC across all models. High WSS acting on the aortic valve is known to promote fibrotic remodeling around the commissures, potentially leading to restricted leaflet mobility and the development or progression of AI [5].

Previous studies using idealized aortic models have simulated the influence of graft position and angle. Gu et al. showed that graft implantation 2 cm from the STJ produced high WSS on the valve leaflets, and that azimuthal positioning altered which cusp received the greatest stress [6]. Wang et al., using fluid–structure interaction, reported substantial WSS on the cusp opposite the graft at an angle of 135° [7]. Iizuka et al. found that larger outflow graft-to-aorta (O-A) angles correlated with AI progression in LVAD patients [8], and their animal study demonstrated that obtuse angles worsened AI hemodynamics and reduced coronary flow [9].

Our results similarly demonstrate that greater outflow graft angles increase WSS, particularly on the LCC—located opposite the anastomosis when the azimuthal angle is fixed at 90°. Although shallow outflow graft angles may reduce adverse WSS, moving the anastomosis more distally is not always feasible in smaller-bodied patients, such as many in the Japanese population.

Surgical management of post-LVAD AI remains controversial. Park’s stitch—a coaptation stitch applied to the nodules of Arantius—has shown favorable outcomes [10, 11]. However, in cases where high WSS is chronically applied to the leaflets, the long-term durability of such repairs may be limited. Valve replacement may be required when leaflet preservation is not feasible. In our Case 1, the patient developed moderate-to-severe AI directed toward the anterior mitral leaflet, consistent with WSS elevation predicted at the LCC. Pathology revealed lymphocytic inflammation and degenerative changes consistent with mechanical stress induced by LVAD flow. During follow-up periods of 5 years (Case 2) and 4 years (Case 3), no coronary events, worsening of aortic insufficiency, or thromboembolic complications were observed. The clinically implanted outflow graft angles were 59.1° in Case 1, 50.4° in Case 2, and 50.8° in Case 3(Fig. 6). In Case 1, the outflow graft angle was the steepest among 3 cases, and aortic insufficiency requiring surgical intervention developed. In other 2 cases with shallower outflow graft angle, aortic insufficiency was not observed. Therefore, the variation of the outflow graft angle may have partly contributed to the different course in the real clinical setting. However, further studies are needed to derive optimal outflow graft angle by predicting the development of valve insufficiency using WSS on the valve cusp.

Coronary flow behavior in LVAD patients has been described in both clinical and large-animal studies [12–14], but CFD-based analyses remain scarce. In this study, shallower outflow graft angles reduced coronary flow and velocity. While reducing WSS on the aortic valve may be desirable, excessively shallow angles risk compromising coronary perfusion. We also clarified that actual coronary vascular resistance is influenced by ventricular pressure, coronary vascular bed resistance, and aortic valve opening and closure, and that the present findings should be interpreted within the context of the simplified modeling assumptions used in this study. Reduced root flow velocity may additionally increase the risk of aortic root thrombosis [15]. Although the present analysis was performed under simplified conditions, a shallower outflow graft angle may reduce coronary blood flow; however, it is uncertain whether such a reduction is sufficient to contribute to myocardial ischemia and coronary artery thrombosis.

Because CFD simulations based on preoperative CT can be performed with relative ease, such patient-specific analyses may help optimize graft angle selection and evaluate the need for prophylactic aortic valve procedures. Several reports have already begun to incorporate CFD into surgical planning [5, 6], suggesting that this approach may improve long-term outcomes in LVAD recipients.

### Limitations

All models assumed rigid arterial walls, and vessel compliance was not included.

The aortic valve was assumed to remain closed under continuous-flow conditions; pulsatile cases with partial valve opening were not evaluated.

Blood viscosity may differ from the values used due to anticoagulation therapy in LVAD patients. Another limitation of this study is the small sample size, consisting of only three cases. While incorporating dynamic aortic valve motion into CT-based CFD simulations remains technically challenging, this approach allows flexible geometric modification of static models and systematic evaluation under different conditions. However, the present model is simplified, and further analyses using a four-dimensional, time-dependent CFD framework would be warranted.

## Conclusions

Shallower outflow graft angles were associated with reduced WSS on the aortic valve. Conversely, steeper angles increased WSS—particularly on the LCC—which may contribute to valve remodeling and AI progression. Given the relative ease of performing patient-specific CFD simulations, this approach may help determine optimal outflow graft orientation for individual LVAD patients.

## References

[1] Slaughter MS, Rogers JG, Milano CA, et al. Advanced heart failure treated with continuous-flow left ventricular assist device. N Engl J Med. 2009;361:2241–51. 19920051 10.1056/NEJMoa0909938

[2] Nakatani T et al. Registry report on heart transplantation in Japan. Circ J. 2019.

[3] Pak SW, Uriel N, Takayama H, et al. Prevalence of de Novo aortic insufficiency during long-term support with a continuous-flow LVAD. Circulation. 2010;122:256–63. 10.1016/j.healun.2010.05.018 20619680

[4] Cowger J, Pagani FD, Haft J, et al. Aortic valve insufficiency in LVAD patients: pathophysiology, clinical impact, and management. J Heart Lung Transpl. 2015;34(11):1495–502.

[5] Karmonik C, Partovi S, Loebe M, et al. Computational fluid dynamics in patients with continuous-flow left ventricular assist device support show hemodynamic alterations in the ascending aorta. J Thorac Cardiovasc Surg. 2014;147(4):1326–33. 24345553 10.1016/j.jtcvs.2013.09.069

[6] Gu Z, Zhang Z, Huang K, et al. The impact of left ventricular assist device outflow graft positioning on aortic hemodynamics: improving flow dynamics to mitigate aortic insufficiency. Biomimetics. 2023;8:465. 37887596 10.3390/biomimetics8060465 PMC10604423

[7] Wang W, Li X, Zhang J, et al. The Biomechanical effect of the O–A angle on the aortic valve under left ventricular assist device support: a primary fluid–structure interaction study. J Thorac Dis. 2024;16(12):8620–32. 39831250 10.21037/jtd-24-1650 PMC11740063

[8] Iizuka K, Hatakeyama T, Tsutsui H, et al. Outflow graft anastomosis site design could be correlated to aortic valve regurgitation under left ventricular assist device support. J Artif Organs. 2018;21:150–5. 29164425 10.1007/s10047-017-1006-1

[9] Iizuka K, Hatakeyama T, Tsutsui H, et al. The angle of the outflow graft to the aorta can affect recirculation due to aortic insufficiency under left ventricular assist device support. J Artif Organs. 2018;21:399–404. 30039455 10.1007/s10047-018-1064-z

[10] Hynds MA, Hall SA, Silvestry SC, et al. Medium-term outcomes of concomitant aortic valve repair in patients with continuous-flow left ventricular assist device. J Thorac Cardiovasc Surg. 2017;154(3):808–17. 10.1016/j.jtcvs.2024.05.016 38802043

[11] Park SJ, Liao KK, Segurola R, et al. Management of aortic insufficiency in patients with left ventricular assist devices: a simple coaptation stitch method (Park’s stitch). J Thorac Cardiovasc Surg. 2004;127(1):264–6. 14752440 10.1016/s0022-5223(03)01301-1

[12] Ootaki Y, Kamohara K, Akiyama M, et al. Phasic coronary blood flow pattern during continuous-flow left ventricular assist support. Eur J Cardiothorac Surg. 2005;28(5):711–6. 16198117 10.1016/j.ejcts.2005.08.008

[13] Tansley P, Birks EJ, Owen CH, et al. Effect of left ventricular assist device combination therapy on myocardial blood flow in patients with end-stage dilated cardiomyopathy. J Heart Lung Transpl. 2004;23(12):1359–67. 10.1016/j.healun.2003.09.005 15539127

[14] Ambardekar AV, Demos DS, Lee S, et al. Coronary artery remodeling and fibrosis with continuous-flow left ventricular assist device support. Circ Heart Fail. 2018;11(5):e004491. 29724722 10.1161/CIRCHEARTFAILURE.117.004491 PMC5941935

[15] Fried J, Farrar DJ, Khalpey Z, et al. Aortic root thrombosis in patients supported with continuous-flow left ventricular assist devices. J Heart Lung Transpl. 2018;37:1425–32. 10.1016/j.healun.2018.07.012 PMC6240485 30241886

